# The Role of Gut Barrier Dysfunction and Microbiome Dysbiosis in Colorectal Cancer Development

**DOI:** 10.3389/fonc.2021.626349

**Published:** 2021-04-15

**Authors:** Flavia Genua, Vedhika Raghunathan, Mazda Jenab, William M. Gallagher, David J. Hughes

**Affiliations:** ^1^ Cancer Biology and Therapeutics Laboratory, Conway Institute, School of Biomedical and Biomolecular Sciences, University College Dublin, Dublin, Ireland; ^2^ College of Literature, Sciences, and the Arts, University of Michigan, Ann Arbor, MI, United States; ^3^ Section of Nutrition and Metabolism, International Agency for Research on Cancer (IARC-WHO), Lyon, France

**Keywords:** colorectal cancer, dysbiosis, biomarkers, colonic mucosal barrier, microbiome

## Abstract

Accumulating evidence indicates that breakdown of the+ protective mucosal barrier of the gut plays a role in colorectal cancer (CRC) development. Inflammation and oxidative stress in the colonic epithelium are thought to be involved in colorectal carcinogenesis and the breakdown of the integrity of the colonic barrier may increase the exposure of colonocytes to toxins from the colonic milieu, enhancing inflammatory processes and release of Reactive Oxygen Species (ROS). The aetiological importance of the gut microbiome and its composition – influenced by consumption of processed meats, red meats and alcoholic drinks, smoking, physical inactivity, obesity - in CRC development is also increasingly being recognized. The gut microbiome has diverse roles, such as in nutrient metabolism and immune modulation. However, microbial encroachment towards the colonic epithelium may promote inflammation and oxidative stress and even translocation of species across the colonic lumen. Recent research suggests that factors that modify the above mechanisms, e.g., obesity and Western diet, also alter gut microbiota, degrade the integrity of the gut protective barrier, and expose colonocytes to toxins. However, it remains unclear how obesity, lifestyle and metabolic factors contribute to gut-barrier integrity, leading to metabolic disturbance, colonocyte damage, and potentially to CRC development. This review will discuss the interactive roles of gut-barrier dysfunction, microbiome dysbiosis, and exposure to endogenous toxins as another mechanism in CRC development, and how biomarkers of colonic mucosal barrier function may provide avenues for disease, prevention and detection.

## Introduction

Colorectal cancer (CRC) is the second leading cause of cancer related death and the third most commonly diagnosed in the world, with 1.8 million new cases in 2018 (1). Improvements in methods and screening programs, such as immunochemical faecal occult blood tests (FIT) and colonoscopy, have reduced mortality rates thanks to the detection and surgical removal of pre-cancerous colorectal adenomas (CRAs) or early-stage cancers (2). Unfortunately, despite increased screening strategies, disease diagnosis is still more often at advanced stages and is associated with poorer prognosis. Accumulating evidence suggests that genetic susceptibility, environmental exposure, metabolic dysfunction, immune and inflammatory factors, microbiome composition and breakdown of gut barrier integrity play major roles in CRC aetiology (3). While most CRCs are considered sporadic, up to 35% is attributed to inherited susceptibility (4). High-penetrance germline mutations in mismatch DNA repair (*MMR*) genes and in Wnt *(APC)*, TGFbeta/BMP signalling pathways (e.g., *MADH4, SMAD4, BMPR1A*) predispose to hereditary CRC syndromes (4). However, these mutations only account for about 6% of CRC while the inheritance of low-risk variants contribute a larger proportion of the genetic factors implicated in CRC development (5). Thus far, genome-wide association studies (GWAS) have identified around 100 loci associated with sporadic CRC, in or proximal to known CRC-related genes and pathways (e.g., *BMP2, BMP4, SMAD7, CCND2, GREM1*) and in genes not previously linked to CRC *(CDKN1A, EIF3H, TPD52L3, ITIH2, LAMA5,* and *LAMC1*) (6, 7). Modifiable lifestyle habits likely explain the worldwide heterogeneity in CRC incidence rates (5). It is well established that increased consumption of processed meats, red meats and alcoholic drinks, smoking, physical inactivity, obesity and adult attained height increase the risk of CRC development, while consumptions of fibres and calcium, and vitamin D levels are associated with a reduced risk (8). Furthermore, although age represents one of the risk factors for CRC, over the recent decades, the incidence rate for those under 50 years old has increased (9), underlying the relevant role of dietary and lifestyle environmental factors in CRC aetiology in addition to genetic predisposition (3).

Thus, the potential preventability of CRC depends on detailed understanding of its aetiology and interactive underlying mechanisms of development.

The role of the gut barrier is being increasingly recognized as pivotal to health and its dysregulation associated with a broad range of diseases, including celiac disease inflammatory bowel disease (IBD) and CRC (10–13). Metabolites derived from the diet can interact with the intestinal epithelium causing stimuli that may directly induce structural damage in epithelial cells and activate pro-carcinogenic signalling pathways, or by indirectly interacting with the gut microbiota (14).

Experimental and observational evidence suggests that the microbiome and its interactions with the human host are involved in most of the biological processes that regulate health and disease (15). The role of dietary and lifestyle factors in impacting microbiome composition has been widely discussed in the literature and it is known that they can affect the relationship between host and microbiome, causing alterations in gut barrier function and immune response (3, 16).

Recently, research has focused on whether disturbance in the balance of commensal microbial species may be carcinogenic and could provide a mechanistic link between dietary and lifestyle aetiological risk factors in CRC development (17). A direct role of the microbiota in carcinogenesis includes infection with Human Papilloma Virus (HPV), *Helicobacter pylori* (*H. pylori*) and Hepatitis B/C virus as the main aetiological risk factors for cervical, gastric and liver cancer development, respectively (18–20). While evidence suggests that microbes in the colonic lumen have the capacity to be carcinogenic given access to colonocytes, they must circumvent barrier functions within and around the intestinal cells (3, 11). Based on the observation of the “leaky gut” in patients with intestinal diseases and the dysbiosis associated with the development of several chronic inflammatory disorders and CRC (21, 22), the study of the colonic barrier status and the markers associated with its damage may aid detection of early stages of intestinal disorders, including CRC. The examination of markers of colonic mucosal barrier dysfunction throughout the development of CRAs and CRCs may help to elucidate the dynamic interaction between the colonic epithelium and commensal bacteria in disease and health status.

In this review, we discuss the dynamic role of colonic mucosal barrier dysfunction (focusing on the mucus layer and epithelial cell lining components of the gut barrier), microbiome dysbiosis, and exposure to endogenous toxins as co-factors in CRC development. We also summarize the latest findings regarding the protein and metabolite markers of colonic mucosal barrier integrity and how these may provide new insights into CRC pathogenesis and as biomarkers of disease detection and prevention.

## Microbiome Dysbiosis and the Gut Epithelial Barrier in Colorectal Cancer Development

Recent compelling evidence in both animal models and human studies suggest that infections by bacterial pathogens, commensal microbial dysregulation, and resultant alterations in microbial products are involved in CRC development (17, 23–25). Interactions of Western-type lifestyle habits (e.g., obesity, unhealthy diet), host genetics and immune responses are thought to induce microbiome changes, eventually leading to bacterial dysbiosis and shift the balance of metabolic function from beneficial to detrimental (17, 24).

The gut barrier is a dynamic and complex environment and acts as a physical and chemical barrier that suppresses the access of pathobionts, antigens and other invasive bacteria into the host (26). The outer mucus layer contains the commensal bacteria that produce antimicrobial proteins and secretory immunoglobulin A (IgA). In the lumen, bacteria and antigens are degraded by the action of gastric, pancreatic, and biliary juice while commensal bacteria produce bacteriocins, modify the pH of the luminal content, and compete for nutrients to inhibit the colonization of pathogens (14, 27). In the epithelium layer, epithelial cells contain tight junction complexes critical for sealing paracellular spaces between colonocytes, maintaining selective permeability and barrier integrity (28, 29). Furthermore, epithelial cells can transport the luminal content and produce antimicrobials to eliminate microorganisms and potentiate the action of monocytes and macrophages (27). The lamina propria contains cells of the immune and adaptive response able to secrete immunoglobulins and cytokines (27). The maintenance of a functional colonic mucosal barrier is essential to guarantee a healthy condition and the luminal confinement of bacteria is one of the key roles of the gut mucosa. Over time, the protective capacity of the colonic mucosal barrier is affected allowing greater bacterial translocation and entry of toxic microbial products, such as pro-inflammatory endotoxins and bacterial metabolites, across the colonic epithelium (24, 30). These exposures may cause localized inflammation and the release of reactive oxygen species (ROS) within colorectal tissues, which may be causative or promotive of neoplastic processes. However, it remains unclear to what extent lifestyle factors impact these processes and whether these events are involved in CRC aetiology, or are consequences of the disease process (5, 30–33). Saus et al. define “driver” bacteria as those that conduct the colonic tissue damage, and “passengers” for those that merely profit from the microenvironment altered by the disease (34). Damage to the gut epithelial barrier mediated by microbes may promote carcinogenesis due to production of bacterial derived genotoxins, e.g., colibactin, and decreasing beneficial bacterial metabolites, such as short chain fatty acids (SCFA) (35, 36). SCFAs help to maintain gut barrier function and it has been shown that enhanced butyrate production may preserve the gastrointestinal epithelial lining through increasing expression of tight junction proteins (37). The breakdown of colonic barrier integrity and functionality impact colonic permeability facilitating microbial encroachment towards the colonic epithelium and greater exposure to toxic, mutagenic, and carcinogenic compounds from the colonic milieu, and bacterial translocation into the epithelium. Thus, there appears to be an intrinsically key relationship between the microbiome and the gut barrier in maintaining a healthy colorectal tract.

Although the definition is not well established and the composition of the microbiome varies among individuals (38), a healthy gut microbiome might be identified as a bacterial community residing in the gastrointestinal tract able to conserve defined functional genes in physiologic conditions (39). In the intestinal microbiome in the absence of evident disease, *Bacteroides* and *Firmicutes* represent a major part of the bacteria in the gut while phyla such as *Proteobacteria, Verrucomicrobia, Actinobacteria*, *Cyanobacteria* and *Fusobacteria* are present in lower abundance (40). Any shift in gut microbial composition might reduce or inhibit the beneficial effects of the physiological functions of commensal microbes and impact the health/disease status of the host (41). Use of antibiotics from after one day of treatment can reduce microbiome diversity (42). The composition of the microbiome can be also impacted by host genetic variation such as in the *VDR* gene, encoding the vitamin D receptor (43) or the *NOD2, LCT* and *MUC2* genes, implicated in the regulation of immune response and secretion of anti-bacterial compounds (44), genetic regulation of *Bifidobacterium* abundance (45) and mucin secretion in the gastrointestinal tract (46). Variations in *FUT2*, a gene regulating the expression of histo-blood group antigens on the gastrointestinal mucosa (47), have been associated with differential interactions with intestinal bacteria and also with CRC risk (6). Furthermore, altered expression of DNA repair or immune-inflammatory pathways, such as innate immunity genes, including *MyD88*, *TLR4* and *TLR5*, have been associated with changes in the microbiome composition (48).

Several research groups have evaluated microbiome components as CRC screening biomarkers, employing qPCR of extracted DNA from either stool, including secondary use of stool based diagnostic tests (e.g., FIT), or tumour tissue samples, to associate the relative abundance of bacterial species with colorectal lesions. Bacterial species most often observed to be over-represented in CRC from these studies include *Fusobacterium nucleatum (Fn), Enterococcus faecalis, Streptococcus gallolyticus* (*SGG)*, enterotoxigenic toxin producing *Bacteroides fragilis (ETBF)*, and *Porphyromonas* species (49–53), while genera such as *Roseburia, Ruminococcus, Clostridium, Faecalibacterium* and *Bifidobacterium* are generally depleted in CRC patients (54–56). However, these study designs cannot directly address the issue of causality, and their variable results are likely due to differing study designs, sample sizes, limited or no data on major confounders, and assay differences (57). Experimental data suggest that microbes such as p*ks* (polyketide synthase)+*Escherichia coli (pks+Ec), ETBF*, *Fn*, and *SGG*, damage the gut-barrier lining and colonocyte DNA, increase proinflammatory cytokines and oxidative factors, and produce potentially carcinogenic toxins (17, 24, 58–61).

The evolving discipline of molecular pathological epidemiology (MPE) offers a powerful approach to explain the interpersonal susceptibility to carcinogenesis and to clarify the role of microbial variation and dysbiosis in the development and progression of tumour anatomical sub-sites within the colorectum and by tumour molecular sub-type features (e.g., inherited *MMR* mutations, somatic mutations such as in the *KRAS*, *APC*, *BRAF* and *PIK3CA* genes, microsatellite instability, epigenetic modifications) (62). Using taxon-specific models, a microbial GWAS study focused on the genetic contribution to microbiome variations and heritability of microbial taxa and identified genetic associations involving multiple microbial traits (63). Demonstrating the utility of such data, a recent study, using the random forest algorithm, established a novel CRC predictive model able to distinguish cancer from healthy subjects based on gut microbial single nucleotide variant markers (64). Using such integrative, multi-omic approaches that combine tumour molecular features, metagenomic, microbiome/human metabolomics, proteomics and nutrigenomics, MPE can add novel insights into the tumour pathology and host/microbiome interactions, potentially providing microbiome-modulating strategies for cancer prevention and treatment (65, 66).

## Gut Epithelial Barrier Function and Colorectal Cancer Development

In the ‘leaky-gut’ hypothesis, there is a loss of epithelial integrity with bacterial translocation across the gut-barrier and detection by the immune system. Thereupon, the activation of cells of the innate and adaptive immune system in the lamina propria, including phagocytes and lymphocytes, are triggered to protect the gut tissue from further microbial damage (30). Commensal microbes support barrier function via colonization resistance, inhibition of certain virulence factors of pathogens, instigating immune responses and pH changes to control pathogens, and secreting antimicrobials to exclude pathogens from epithelial cells (67). The status of the mucus layer is balanced between the turnover of the goblet cells in the intestinal epithelium producing the mucus through the action of mucin 2 glycoprotein (MUC2) (30, 68), and degradation by gut bacteria. This is likely protected by interaction between dietary fibre and gut microbiota, as recently shown for mucus and barrier integrity in a mouse model, where mucus in synthetic microbiota-colonized fibre free diet mice was five to six times thinner than colonized mice fed the fibre rich diet (68). Murine models have demonstrated that genetic ablation of *Muc2* brings bacteria into close contact with the epithelium, leading to inflammation and colon cancer (69). Strong evidence confirmed that different bacteria, including *E. coli, Enterococcus faecalis, Bacteroides vultagus* and several *Fusobacterium* strains isolated from Crohn’s disease or ulcerative colitis patients were found to invade epithelial cells *in vitro* (70–72). These findings have been confirmed *in vivo*. The ability of *Fn* to invade epithelial cells via FadA binding to CDH1 (E- cadherin) and to promote inflammation and oncogenic signalling has been demonstrated using HCT116 xenograft mice (73) and *Fn* infection in an Apc^Min/+^ mouse model lead to the generation of a proinflammatory microenvironment (59). It has been also observed that the Bft toxin from ETBF induces colitis and disrupts CDH1 junction, activates CTNNB1 (beta catenin) signalling, and induces IL8 secretion in murine colonic epithelial cells (CECs) (74, 75). The *E. coli* pks+ strain promoted invasive carcinoma in an azoxymethane (AOM)-treated IL-10^−/−^ mouse model, while the deletion of the pks genotoxic island from the same strain decreased tumour multiplicity and invasion in AOM/IL10^−/−^ mice (76). In addition to these bacteria, the gut symbiont *Akkermansia muciniphila (A. muciniphila)* has been implicated in the modulation of mucus layer thickness in the gut barrier (77, 78). Moreover, it has been shown that a recombinant protein Amuc_1434* - derived from *A. muciniphila* and expressed in an *E.coli* prokaryote cell system-inhibited the proliferation of CRC cell lines (LS174T) through the degradation of Muc2 (79). As expression of MUC2 in CRC tissue has been shown to be significantly increased compared to the normal mucosal control (80), *A. muciniphila* may inhibit development and progression of CRC through the restoration of the normal Muc2 level (79). Another study observed that the outer membrane protein Amuc_1100 increased the development of trans epithelial electrical Resistance in Caco2 cells, which indicate a role in the maintenance of gut barrier integrity (81). Animal studies confirmed that the recombinant Amuc_1100 protein improves gut barrier functionality and restrains the development of high-fat diet-induced obesity in mice (82). Furthermore, lower levels of *A. muciniphila* have been observed in faecal microbiota of colitis patients (83, 84), and biopsies of intestinal mucosa from IBD-patients compared to controls (85), further indicating the protective role of this symbiont in gut intestinal health.

**Figure 1:**
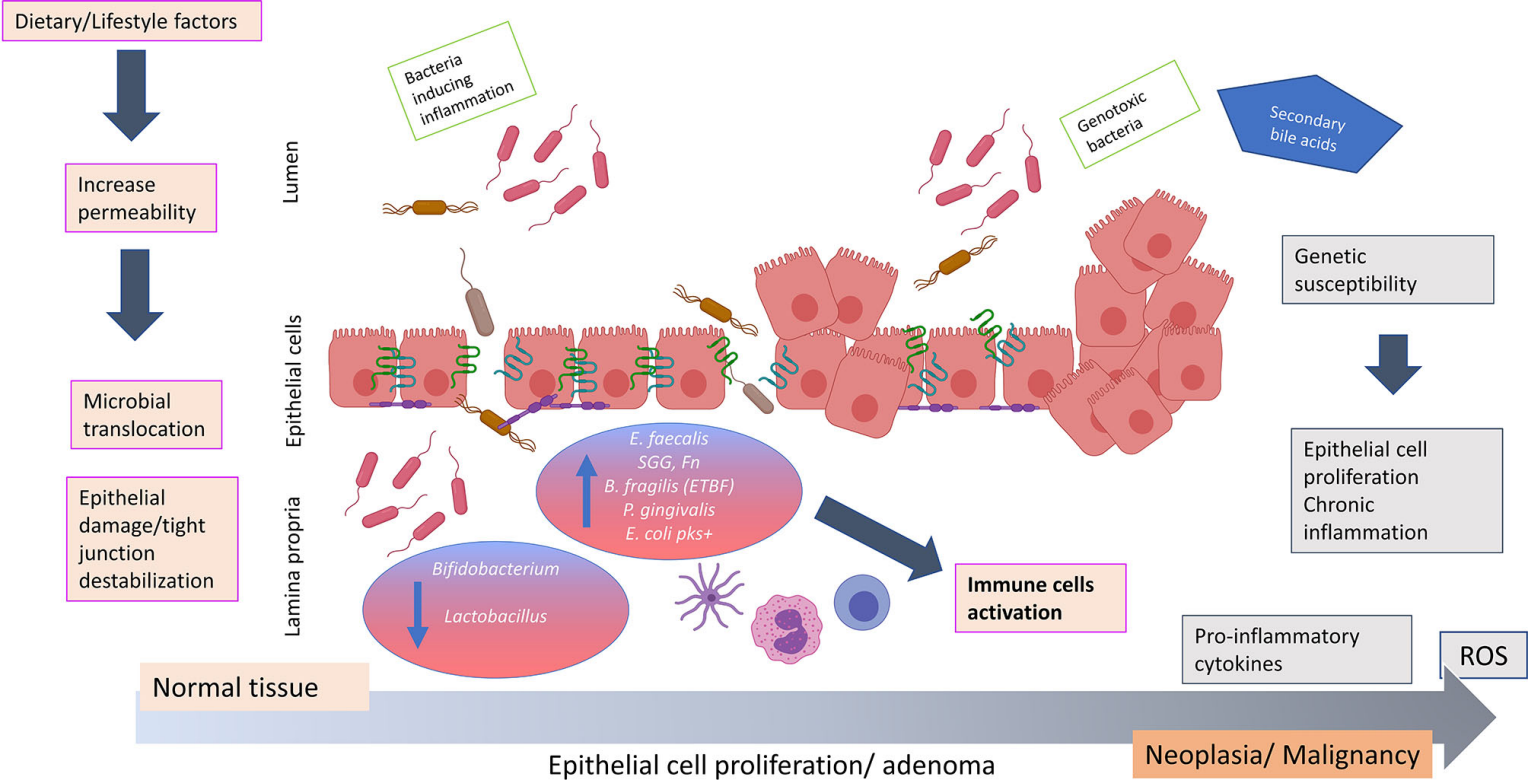
Hypothesis of how bacterial translocation across a weakened colonic mucosal barrier may promote colorectal carcinogenesis. The impaired of barrier integrity aggravated by dietary/lifestyle factors contributes to an important shift in the microbiota composition. Commensal bacteria may acquire invasiveness properties and disturb the epithelial structure causing the passage of bacterial strains such as *Fusobacterium nucleatum, Bacteriodes fragilis, Streptococcus gallolyticus* subspecies *gallolyticus, Porphyromonas gingivalis, Escherichia coli* pks+ and *Enterococcus faecalis*. The invasiveness properties of these microbes due to virulence factors may impair the gut barrier integrity and cause epithelial damage and tight junction destablization. This aggravates bacterial translocation creating an environment favorable to disease promoting (potentially exacerbated in individuals of contributing genetic predisposition), resulting in the production of pro-inflammatory cytokines (IL3, IL10, IL6, TNFα) and release of reactive oxygen species. These events may potentiate chronic inflammation, uncontrolled epithelial cell proliferation and colorectal neoplasia formation. ROS, reactive oxygen species; IL, interleukin; TNF, tumor necrosis factor; SCFA, short chain fatty acids; *E. faecalis, Enterococcus faecalis; SGG, Streptococcus gallolyticus subspecies gallolyticus*; *Fn, Fusobacterium nucleatum*; *B. fragilis, Bacteriodes fragilis*; ETBF, enterotoxigenic toxin producing *Bacteriodes fragilis*; *P. gingivalis, Porphyromonas gingivalis*; *E. coli* pks+, *Escherichia coli* pks+. The figure was created using BioRender.com.

It is well established that inflammation is a key environmental trigger that affects microbial composition and chronic inflammation is a hallmark of intestinal carcinogenesis (86). However, it is unclear whether inflammation is promoted by microbial dysbiosis imprinted early on by host genetics and/or diet/lifestyle, or if dysbiosis arises due to advancing inflammatory grades (58). One hypothesis for the primary role of microbial dysbiosis associated with a leaky gut in the development of several diseases, including CRC, is that the weakness of the gut barrier causes a shift in the microbial community whereon the commensal bacteria within the epithelial cells may become pathogenic by acquiring virulence factors (29). This can disturb the epithelial structure and destabilize tight junction, causing the passage of bacterial strains. Both the bacterial invasiveness and the damage of the barrier contribute to a bacterial translocation and immune hyper activation in colonic mucosa. This condition could induce a further shift in the microbiota composition which may cause chronic inflammation and CRC onset (29) (**Figure 1**). Progression from damaged gut barriers through increasingly dysplastic neoplasms and finally tumorigenesis may be exacerbated in ‘vulnerable’ hosts, such as in obese or diabetic individuals or those with genetic predisposition to CRC (17).

## Markers of Gut Barrier Damage and Their Potential Application in Early Detection of Colorectal Cancer

Together, the existing evidence implies a key mechanistic relationship between gut barrier integrity and microbiome composition and metabolism with colonic health (3). Indeed, levels of proteins implicated in maintaining gut barrier function have been found to be altered in inflammatory intestinal disease and CRC (87, 88). Therefore, protein markers of gut barrier function might be used as biomarkers for the early detection of CRC or stratification of the malignant potential of adenomas.

In this section we discuss promising candidate biomarkers of intestinal barrier integrity relevant to CRC. These may be classified, according to their mechanism of action at the intestinal lining, as direct or indirect biomarkers of gut-barrier functionality (**Table 1**).

**Table 1:** Candidate biomarkers for gut barrier damage in colorectal carcinogenesis.

	Candidate biomarkers	Putative function	Evidence as biomarkers	Reference
	D-lactate and diamine oxidase (DAO)	Intestinal permeability	Plasma levels of D-lactate and DAO were found to be increased in CRC patients (n=53) compared to control (n=45)	(89)
Direct measurement of intestinal damage	iFABP	Intestinal permeability	Plasma concentration levels increased in patients with severe ulcerative colitis (n = 42)	(90)
	Zonulin	Intestinal permeability	Faecal levels increased in patients with Crohn’s disease (n=37) compared to control (n=40) Mice gavaged with zonulin showed increased both small intestinal and gastroduodenal permeability compared with bovine serum albumine-treated controls	(91) (92)
	ZO1	Tight Junction integrity	Lower levels in colonic mucosa of IBD patients (n=50) compared to controls (n=31) Knock out in mouse epithelial cell lines result in a loss of tight junction strands	(93) (94)
	CLDN1	Tight Junction integrity	CLDN1 expression was increased in both high-grade dysplasia and ulcerative colitis-associated CRC tissue (n=6) when compared with ulcerative colitis and normal tissue (n=39) The upregulation of claudin-1 in transgenic mice induces MMP-9 and p-ERK signalling to activate Notch-signalling pathway	(95) (96)
	JAM2	Tight Junction structure	JAM-2 expression was decreased in colorectal cell lines and CRC tissue (n=94) compared to controls (75)	(97)
	MLCK	Tight Junction regulation	Upregulated in IBD in human intestinal resection and biopsy specimens	(98)
Indirect measurement of intestinal damage	LPS and flagellin	Intestinal permeability	Serum LPS- and flagellin-specific immunoglobulin levels positively associated to CRC risk among men in a prospective cohort study. A borderline statistically significant inverse association was observed for women. Levels of LPS were found to be increased in CRC patients (n=53) compared to control (n=45)	(99) (89)
	Soluble CD14	Response to LPS exposure	Increased TLR4-CD14 expression were found in Caco-2 cell lines Plasma levels of sCD14 were positively associated with a Western diet (n=1198) in a cross-sectional study	(100) (101)
	LPS-binding protein (LBP)	Exposure to LPS	Levels of serum LBP were higher in CRC patients with cachexia (n=74) than in CRC patients without cachexia (n=78)	(102)
	Cytokine markers (e.g., IFNgamma, IL10, IL12p70, IL13, IL1beta, IL2, IL4, IL6, IL8, TNFalfa)	Inflammation	Early barrier loss and activation of IL23/IL17-driven tumour-elicited inflammation act additively and sequentially to genetically controlled events that govern CRC development and progression in Apc^F/WT^mice IFNgamma and TNFalfa alter barrier properties of the intestinal epithelium increasing epithelial paracellular permeability human in cell lines	(103) (104)
	Short chain fatty acids (SCFA)	Intestinal permeability	A cross sectional study observed that faecal levels of SCFA was significantly lower in CRC patients (n=19) compared to healthy control (n=16) Levels of bacteria producing SCFA assessed in stool samples were lower in CRC patient (n=15) compared to control (n=12)	(105) (106)
	Secondary bile acids	Intestinal permeability	Apc ^min/+^ mice treated with cholic acid (n=10) showed an increased intestinal permeability compared to control (n=10)	(107)
	Vitamin D and VDR	Intestinal damage	*In vivo* model of infectious colitis showed that vitamin D deficiency increased colonic hyperplasia and epithelial barrier dysfunction	(108)
	Calprotectin	Inflammation	Elevated faecal calprotectin levels associated with intestinal inflammation and IBD	(109)

CRC, colorectal cancer; iFABP, intestinal fatty acid binding protein; LPS, Lipopolysaccharide; ZO1, Zonula Occludens 1; IBD, irritable bowel syndrome; CLDN1, claudin 1; CLDN2, claudin2; JAM2, Junctional adhesive molecule 2; MLCK, myosin ligase chain kinase.

### Direct Gut Barrier Damage Markers

Proteins described within this section appear to be promising candidate biomarkers of barrier functionality as their position in the enterocytes and in the tight junction complex make them possibly more mechanistically linked to colorectal carcinogenesis mediated by gut barrier damage.

These proteins include the intestinal fatty acid binding protein (iFABP), a low molecular mass (~14-15 kDa) intracellular protein present in the epithelial cells of the intestinal mucosal layer (110). The location of iFABP facilitates its leakage into the circulation from enterocytes during intestinal damage, and results in upregulation of this protein. iFABP as a marker of enterocyte damage has been described in irritable bowel disease, necrotising colitis and coeliac disease (111). Plasma levels of iFABP were observed to be higher in patients with a severe form of ulcerative colitis compared to those with a mild form of the disease, and higher serum levels of iFABP were reported in celiac disease patients who had increased intestinal permeability compared to healthy controls (112), underlying that this protein might be used as an indicator of an extended inflammatory process (90). Therefore, iFABP appears to be a useful candidate to evaluate gut barrier damage and inflammation in CRC (113).

Another protein that has been proposed as a direct biomarker of gut barrier integrity is zonulin [prehaptoglobin-2, (92)], a protein involved in the regulation of tight junction (114). It has been reported that zonulin is upregulated during the acute phase of celiac disease (115). Increased serum and faecal levels of zonulin were also found in Crohn’s patients compared to controls (91). Furthermore, an *in vivo* study observed that mice gavaged with zonulin showed increases in both small intestinal and gastroduodenal permeability compared with bovine serum albumin-treated controls (92). Asmar et al (2000) indicated that the exposure of mammalian small intestine to either pathogenic or non-pathogenic bacteria induces intraluminal zonulin secretion and increased intestinal permeability (116). Release of zonulin after bacterial exposure has been demonstrated in rat and human intestinal cell lines (IEC6 and Caco-2, respectively), and may facilitate the flushing out of microbes and toxins, participating in the innate immune response of the host to counter intestinal bacterial colonisation (117).

As it is well established that a “leaky” barrier characterized by increased tight junction permeability is a hallmark of pathological conditions including CRC, proteins involved in tight junction structure represent an effective target for the evaluation of gut barrier status. Several tight junction proteins have been implicated in the pathogenesis of gastrointestinal cancers (118). In CRC, claudins (CLDNs) play an important role in the neoplastic transformation of premalignant epithelium tissue as demonstrated in APC^min^ mice, where the overexpression of Cldn1 induced tumour growth and progression (119). Using microarrays analysis in APC^min^ mice, the same study showed that genes regulating mucosal defence (*Muc2, Klf4* and *Tff3*) were downregulated while pro-inflammatory pathways, especially involving IL23/IL17 signalling, were upregulated (119). Expression of Claudin1 (CLDN1) has been shown to be increased in both high-grade dysplasia and ulcerative colitis-associated CRC tissue when compared to ulcerative colitis and normal tissue (95). Transgenic mice overexpressing intestinal Cldn1 have been used to demonstrate consequent induction of MMP9 and pERK signalling and Notch-signalling pathway activation, leading to inhibition of goblet cell differentiation, decreased Muc2 expression and resultant mucosal inflammation (96). The junctional adhesion proteins (JAMs) are involved in the formation of the tight junction, and lower expression of JAM2 has been associated with CRC disease progression, metastasis and poor prognosis, indicating JAM2 as a tumour suppressor (97). Zonula Occludens 1 (ZO1) was revealed to be essential in tight junction strand assembly as its knock out in mouse epithelial cell lines result in a loss of tight junction strands (94). Although ZO1 displacement is not sufficient to cause a barrier defect, the combination with other signalling pathways affecting tight junction structure, may contribute to a rearrangement of junctional complex, impacting on tight junction stability (114). ZO1 activity is regulated via MLCK (myosin light chain kinase) pathway and, thus, this protein represents another interesting target of gut barrier function. Expression of Mlck was reported to be increased in murine models of colitis, resulting in dysregulation of tight junction and a severe loss of epithelial barrier function (120). Furthermore, MLCK was also shown to be up-regulated in IBD patients, implicating its involvement in altered epithelial integrity (98).

A recent study conducted by Liu et al, proposed D-lactate and diamine oxidase (DAO) as indicators to evaluate gut barrier integrity, and reported that their levels were increased in plasma from CRC patients compared to controls (89). D-lactate is produced by bacterial fermentation while DAO is an enzyme mainly produced in the small intestine and involved in the histamine metabolism (121). Both levels of D-lactate and DAO correlated positively with levels of *Fn* and *Enterobacteriaceae* measured by qPCR in CRC stool samples, indicating that abundance of these bacterial species, implicated in CRC, may also reflect gut mucosal barrier dysfunction (89).

### Indirect Gut Barrier Damage Markers

Gut microbiota play a pivotal role in homeostasis by processing nutrients from ingested food and producing numerous metabolites for the human body (107). The influence of diet on the composition of the microbiota and the exposure to metabolites produced by gut bacteria influences the intestinal epithelium and is associated with CRC risk modification (122, 123). Secondary bile acids derive from the modification of primary bile acids by gut commensals such as some *Clostridia* species in the colon (124). Several factors including high dietary fat intake have been found to increase bile acid levels (125). More recently, a study conducted in human colon carcinoma HCT116 cells showed that lithocholic acid (LCA), a secondary bile acid, stimulates IL-8 expression and induces endothelial cell proliferation and tube like formation in the tumour microenvironment, providing the strong evidence for LCA as a tumour promoter in CRC (126). *In vivo* studies in Apc^min/+^ mice demonstrated that deoxycholic acid (DCA) promotes the adenoma-adenocarcinoma transition and DCA‐induced changes to the microbial community promoted intestinal carcinogenesis (127, 128). In 2019, the same authors demonstrated that intestinal permeability was significantly augmented in mice treated with cholic acid and that the relative abundance of *Akkermansia* and *Bacteroides* increased in the treated group compare to the control, suggesting an aggravated intestinal inflammation and impaired gut barrier function (107). In contrast with secondary bile acids, SCFA help to reinforce the colonic gut barrier through increased expression of tight junction proteins (37, 56). A cross sectional study observed that faecal levels of SCFAs were significantly lower in CRC patients compared to control subjects (105) and levels of butyrate producing bacteria, such as *Ruminococcus* spp. and *Pseudobutyrivibrio ruminis*, were found to be lower in stool samples from CRC patients in comparison to controls (106). Epidemiology studies have observed that African Americans have a higher risk of developing CRC in association with a consumption of a high fat diet (129–131), and levels of acetate, butyrate, and total SCFAs have been found to be lower in African Americans than other racial/ethnic groups, indicating a strong link between diet, SCFA levels and hence, CRC risk (132). The proposed protection from CRC development from sufficient intake of micronutrients such as selenium, vitamin D, and zinc is largely attributed to their key roles in redox and immune functioning (133–135). However, this may also be mediated by their links with modification of the microbiome and gut barrier function (136–138). For example, an *in vivo* study has demonstrated that mice supplied with a high selenium diet exhibited an abundance of *Akkermansia* and *Turicibacter* (bacteria implicated in gut barrier protection) compared to those fed with a low selenium diet (137). *In vitro* and *in vivo* studies indicate that the vitamin D signalling pathway is essential for epithelial barrier function through increasing the expression of tight junction proteins (139). Knockout of the *VDR* gene in a Caco2 cell line showed a reduction in tight junction protein abundance and compromised function, while Vdr^-/-^ mice have been shown to develop severe colitis compared to Vdr ^+/+^mice (140). Finally, 1,25-dihydroxyvitaminD3, an active form of vitamin D, was found to inhibit enterohaemorrhagic *E. coli*–induced reduction in transepithelial electrical resistance, preserving paracellular permeability and tight junction structure in Caco2 cell lines (108). Thus, these studies indicate that vitamin D deficiency may increase susceptibility to mucosal damage.

One of the most studied indicators of gut barrier status is lipopolysaccharide (LPS), a large molecule located in the outer membrane of gram-negative bacteria. Increased permeability of the gut barrier leads to extensive translocation of microbes into the lamina propria, which is associated with higher circulating levels of LPS (141). High levels of LPS promotes metabolic endotoxemia, and may contribute to CRC development (142). A related bacterial product is represented by flagellin that acts as target of humoral immune response against infections (143). Measurement of LPS and flagellin is challenging as their presence in blood is transient. Hence, an alternative indirect method of measurement is represented by the evaluation of immunoglobulins anti-LPS and anti-flagellin, whose levels can persist longer in the human body (99). Results from the large European Prospective Investigation into Cancer and Nutrition (EPIC) showed that a combined immune response to LPS and flagellin was associated with a higher CRC risk in men, while a borderline statistically significant inverse association was observed for women (99). This contrasting result by sex might be due to immune system variation, as it is known that women have a stronger humoral/innate immune defence than men (144, 145), or, it could be attributable to a different microbiome composition (146). Levels of LPS were also measured directly in plasma; a recent study observed that levels of LPS were increased in CRC patients compared to controls, and this correlated positively with *Fn* and *Enterobacteriaceae*, indicating raised intestinal permeability in CRC cases (89). Another indicator of the amount of effective LPS present in the body is lipopolysaccharide binding protein (LBP), an acute-phase protein produced in the liver and circulating in the bloodstream (147). LBP is able to bind LPS and amplify the host immune response against it (148). A recent study showed that serum LBP levels were higher in colon cachectic carcinoma than in non-cachectic carcinoma patients, indicating this molecule as a biomarker for cancer progression and cachexia development (102).

A measurement of response to LPS is represented by the cluster of differentiation 14 (CD14) protein critical to instigating the host immune response for preserving gut barrier health. Membrane associated CD14 forms a receptor complex with TLR4 (a pattern recognition receptor) and Myeloid differentiation 2 (MD2) and plays an important role in binding LPS, as well as initiating the immune response. In healthy individuals, LPS remains in the lumen due to proper tight junction seclusion among other barrier functions. An increase in TLR4 under the presence of LPS has been associated with an increase in CD14, both of which correlated with an increase in intestinal tight junction permeability *in vitro* and *in vivo* (100). This increase in permeability further compromises gut barrier function, potentially contributing to carcinogenesis. Furthermore, a cross sectional study observed that plasma soluble CD14 (sCD14) levels tend to increase with a Western diet, underlying the association between dietary and intestinal gut barrier dysfunction (101). Bacterial LPS also activate macrophages exposing CD14 and this evokes an inflammatory response. As a consequence, a large amount of cytokines, such as IFNgamma, are produced and this can stimulate the production of other cytokines, including IL10, IL12, TNFalfa, in a positive feedback loop (149). A study conducted in Apc^f/wt^ mice -in which tumourigenesis was caused by Apc allelic loss showed that early CRC- inducing genetic events may cause local loss of barrier function and entry of microbial products into the tumour microenvironment. This results in activation of IL23-producing myeloid cells, which regulate expression of downstream tumour-promoting cytokines, including IL17 and IL6 (103). Furthermore, it has been demonstrated that TNFalfa and IFNgamma are both elevated in the mucosa of IBD patients and they induce changes in epithelial paracellular permeability, associating with tight junction protein restructuring (104). Numerous studies have showed that inflammatory reactions weaken tight junctions and further aggravate gut barrier damage (150, 151), which may over time lead to carcinogenesis.

It has been hypothesized that increased gut permeability in CRC development might be related to an increased immune response to pathobiont or pathogenic gut bacteria. This has encouraged recent serology screening studies of antibodies against microbes as markers of colorectal neoplasia and of gut barrier damage. For example, a significant association between antibody responses to *SGG* and CRC has been observed in several studies (152, 153). More convincingly, a study within the EPIC observed a positive association of antibody responses to *SGG* proteins with CRC risk in pre-diagnostic serum samples, implicating *SGG* serology as a potential marker for risk of developing CRC (154). Regarding the question of whether *SGG* infects colon tissue via a ‘leaky’ epithelial barrier before or after initiation of tumour development, cell line and mouse model studies of CRC demonstrated that *SGG* actively promotes colon cancer cell proliferation and tumour growth (52). However, as indicated in both EPIC and a recent large, prospective CRC cohort consortium in the USA (using the same analytic technique and laboratory as the contemporaneous European study (154)) concluded that, due to the long development time for colorectal tumours and the sensitivity analyses by follow-up time, *SGG* probably only promotes tumourigenesis after it has already begun (155). The controversy over the potential role of *H. pylori* in CRC has also been addressed in serology studies in EPIC and a larger multi-cohort study from the USA, where the antibody responses to *H. pylori* proteins, specifically HcpC and VacA, were associated in both settings with an increased risk of developing CRC (156, 157). It should be noted that in these serology studies it is impossible to know when infection first occurred, confounding an accurate sensitivity analysis of follow-up time to disease diagnosis.

All together these studies support the possible use of human proteins, such as iFABP and CD14, bacterial markers of endotoxemia and metabolites such as LPS, secondary bile acids, and SCFAs as promising biomarkers to evaluate gut barrier damage and suggest dietary strategies and use of pre-and pro-biotics for CRC prevention and treatment.

## Future Directions

While the role of the colonic mucosal barrier and microbial dysbiosis is increasingly recognised as pivotal in the development of colorectal carcinogenesis, there is only limited information on the use of biomarkers of colonic barrier status in association with CRC initiation and progression (as discussed in this review). Observational and experimental studies, which have thus far been more focused on other gastrointestinal diseases like IBD, are required to further assess these markers in relation to prediction of CRC development risk and progression, and to further define the mechanisms of carcinogenesis in interaction with lifestyle and microbiome factors. Although the state-of-art in CRC detection primarily remain FIT and colonoscopy, blood biomarkers of gut barrier function may have uses for minimally invasive screening applications, including as refinements to improve FIT screening accuracy. The protective role of the colonic mucosal barrier in inflammation, oxidative stress, and microbial translocation, underly the applicability of biomarkers of its dysfunction in CRC prevention and early detection.

The understanding of the interaction between host epithelium and the microbiome will provide novel information for the development of prophylactic and therapeutic interventions for CRC patients. Specifically, the inclusion of lifestyle/dietary modifications to improve gut-barrier functionality and promote gastrointestinal eubiosis could be used in both cancer prevention and patient clinical management settings. Dietary modification with increased fibre intake, alternatives to antibiotics, use of pre- and pro-biotics, and vitamin D supplementation, to restore levels of beneficial microbes such as *Lactobacillus* and *Bifidobacterium*, might rebalance gut dysbiosis and help repair barrier damage in CRC patients. Furthermore, the use of MPE, nutritional epidemiology, and microbial GWAS studies can provide novel strategies to integrate genetic variations and modifiable factors into the study of microbiome composition, gut barrier alterations, and links with tumour molecular sub-types. This multidisciplinary model, together with machine learning approaches to better mine the data, should provide new insight into the microbial-gut barrier nexus for improved personalized CRC prevention and disease treatment strategies.

## Conclusion

The understanding of the interaction between the microbiome and the gut barrier will help to elucidate the pathogenesis of several intestinal diseases, including CRC. In this review, we have outlined candidate screening biomarkers representative of a compromised gut epithelium with a concomitant higher risk of CRC development. Although the applicability of these proteins as markers of intestinal damage relevant to CRC pathogenesis is still uncertain and the subject of current investigation, the use of biomarkers of microbial pathogenicity and gut-barrier status may help CRC prevention, screening, and patient management. Further studies are required to assess the potential of these biomarkers for use in clinical practice.

## Author Contributions

Conceptualization: DH and MJ. Funding acquisition: DH and WG. Writing—original draft: FG, DH, and VR. Writing—review and editing: MJ and WG. All authors contributed to the article and approved the submitted version.

## Funding

WG is supported by Science Foundation Ireland under the Investigator Programme Grant OPTi-PREDICT (15/IA/3104) and the Strategic Partnership Programme Precision Oncology Ireland (18/SPP/3522). Support for this work was also provided by the COST Action CA17118 supported by COST (European Cooperation in Science and Technology, www.cost.eu) to FG and DH. FG is supported by a PhD research scholar award (2018-2022) to DH from the School of Biomedical and Biomolecular Sciences, UCD.

## Disclaimer

Where authors are identified as personnel of the International Agency for Research on Cancer / World Health Organization, the authors alone are responsible for the views expressed in this article and they do not necessarily represent the decisions, policy or views of the International Agency for Research on Cancer / World Health Organization.

## Conflict of Interest

WG is a co-founder and Chief Scientific Officer in OncoMark Limited.

The remaining authors declare that the research was conducted in the absence of any commercial or financial relationships that could be construed as a potential conflict of interest.

## References

[1] Bray F Ferlay J Soerjomataram I Siegel RL Torre LA Jemal A. Global cancer statistics 2018: GLOBOCAN estimates of incidence and mortality worldwide for 36 cancers in 185 countries. CA Cancer J Clin (2018) 68(6):394–424. 10.3322/caac.21492 30207593

[2] Lieberman D Ladabaum U Cruz-Correa M Ginsburg C Inadomi JM Kim LS. Screening for colorectal cancer and evolving issues for physicians and patients: A review. JAMA - J Am Med Assoc (2016) 316(20):2135–45. 10.1001/jama.2016.17418 27893135

[3] Murphy N Moreno V Hughes DJ Vodicka L Vodicka P Aglago EK. Lifestyle and dietary environmental factors in colorectal cancer susceptibility. Mol Aspects Med (2019) 69:2–9. 10.1016/j.mam.2019.06.005 31233770

[4] Peters U Bien S Zubair N. Genetic architecture of colorectal cancer. Gut (2015) 64(10):1623–36. 10.1136/gutjnl-2013-306705 PMC4567512 26187503

[5] Hughes LAE Simons CCJM van den Brandt PA van Engeland M Weijenberg MP. Lifestyle, Diet, and Colorectal Cancer Risk According to (Epi)genetic Instability: Current Evidence and Future Directions of Molecular Pathological Epidemiology. Curr Colorectal Cancer Rep (2017) 13(6):455–69. 10.1007/s11888-017-0395-0 PMC5725509 29249914

[6] Law PJ Timofeeva M Fernandez-Rozadilla C Broderick P Studd J Fernandez-Tajes J. Association analyses identify 31 new risk loci for colorectal cancer susceptibility. Nat Commun (2019) 10(1):2154. 10.1038/s41467-019-09775-w 31089142 PMC6517433

[7] Huyghe JR Bien SA Harrison TA Kang HM Chen S Schmit SL. Discovery of common and rare genetic risk variants for colorectal cancer. Nat Genet (2019) 51(1):76–87. 10.1038/s41588-018-0286-6 30510241 PMC6358437

[8] Vieira AR Abar L Chan DSM Vingeliene S Polemiti E Stevens C. Foods and beverages and colorectal cancer risk: A systematic review and meta-analysis of cohort studies, an update of the evidence of the WCRF-AICR Continuous Update Project. Ann Oncol (2017) 28(8):1788–802. 10.1093/annonc/mdx171 28407090

[9] Arnold M Sierra MS Laversanne M Soerjomataram I Jemal A Bray F. Global patterns and trends in colorectal cancer incidence and mortality. Gut (2017) 66(4):683–91. 10.1136/gutjnl-2015-310912 26818619

[10] Mu Q Kirby J Reilly CM Luo XM. Leaky gut as a danger signal for autoimmune diseases. Front Immunol (2017) 8:598. 10.3389/fimmu.2017.00598 28588585 PMC5440529

[11] Scaldaferri F Pizzoferrato M Gerardi V Lopetuso L Gasbarrini A. The gut barrier: New acquisitions and therapeutic approaches. J Clin Gastroenterol (2012) 46 Suppl:S12–7. 10.1097/MCG.0b013e31826ae849 22955350

[12] Weber CR Turner JR. Inflammatory bowel disease: Is it really just another break in the wall? Gut (2007) 56(1):6–8. 10.1136/gut.2006.104182 17172583 PMC1856658

[13] Irrazábal T Belcheva A Girardin SE Martin A Philpott DJ. The multifaceted role of the intestinal microbiota in colon cancer. Mol Cell (2014) 54(2):309–20. 10.1016/j.molcel.2014.03.039 24766895

[14] Vancamelbeke M Vermeire S. The intestinal barrier: a fundamental role in health and disease. Expert Rev Gastroenterol Hepatol (2017) 11(9):821–34. 10.1080/17474124.2017.1343143 PMC6104804 28650209

[15] Blaser MJ. The microbiome revolution. J Clin Invest (2014) 124(10):4162–5. 10.1172/JCI78366 PMC4191014 25271724

[16] Rinninella E Cintoni M Raoul P Lopetuso LR Scaldaferri F Pulcini G. Food components and dietary habits: Keys for a healthy gut microbiota composition. Nutrients (2019) 11(10):2393. 10.3390/nu11102393 PMC6835969 31591348

[17] Scott AJ Alexander JL Merrifield CA Cunningham D Jobin C Brown R. International Cancer Microbiome Consortium consensus statement on the role of the human microbiome in carcinogenesis. Gut (2019) 68(9):1624–32. 10.1136/gutjnl-2019-318556 PMC6709773 31092590

[18] Dunne EF Park IU. HPV and HPV-associated diseases. Infect Dis Clinics North Am (2013) 27(4):765–78. 10.1016/j.idc.2013.09.001 24275269

[19] Kamangar F Dawsey SM Blaser MJ Perez-Perez GI Pietinen P Newschaffer CJ. Opposing risks of gastric cardia and noncardia gastric adenocarcinomas associated with Helicobacter pylori seropositivity. J Natl Cancer Inst (2006) 98(20):1445–52. 10.1093/jnci/djj393 17047193

[20] El-Serag HB. Epidemiology of viral hepatitis and hepatocellular carcinoma. Gastroenterology (2012) 142(6):1264–73.e1. 10.1053/j.gastro.2011.12.061 PMC3338949 22537432

[21] Klingensmith NJ Coopersmith CM. The Gut as the Motor of Multiple Organ Dysfunction in Critical Illness. Crit Care Clin. (2016) 32(2):203–12. 10.1016/j.ccc.2015.11.004 PMC4808565 27016162

[22] Hiippala K Jouhten H Ronkainen A Hartikainen A Kainulainen V Jalanka J. The potential of gut commensals in reinforcing intestinal barrier function and alleviating inflammation. Nutrients (2018) 10(8):988. 10.3390/nu10080988 PMC6116138 30060606

[23] Gao R Gao Z Huang L Qin H. Gut microbiota and colorectal cancer. Eur J Clin Microbiol Infect Dis (2017) 36(5):757–69. 10.1007/s10096-016-2881-8 PMC5395603 28063002

[24] Tilg H Adolph TE Gerner RR Moschen AR. The Intestinal Microbiota in Colorectal Cancer. Cancer Cell (2018) 33(6):954–64. 10.1016/j.ccell.2018.03.004 29657127

[25] Dahmus JD Kotler DL Kastenberg DM Kistler CA. The gut microbiome and colorectal cancer: A review of bacterial pathogenesis. J Gastrointest Oncol (2018) 9(4):769–77. 10.21037/jgo.2018.04.07 PMC6087872 30151274

[26] Chelakkot C Ghim J Ryu SH. Mechanisms regulating intestinal barrier integrity and its pathological implications. Exp Mol Med (2018) 50(8):1–9. 10.1038/s12276-018-0126-x PMC6095905 30115904

[27] Keita ÅV Söderholm JD. The intestinal barrier and its regulation by neuroimmune factors. Neurogastroenterol Motil (2010) 22(7):718–33. 10.1111/j.1365-2982.2010.01498.x 20377785

[28] Boleij A Hechenbleikner EM Goodwin AC Badani R Stein EM Lazarev MG. The bacteroides fragilis toxin gene is prevalent in the colon mucosa of colorectal cancer patients. Clin Infect Dis (2015) 60(2):208–15. 10.1093/cid/ciu787 PMC4351371 25305284

[29] Yu LCH. Microbiota dysbiosis and barrier dysfunction in inflammatory bowel disease and colorectal cancers: exploring a common ground hypothesis. J Biomed Sci (2018) 25(1):79. 10.1186/s12929-018-0483-8 30413188 PMC6234774

[30] Bhatt AP Redinbo MR Bultman SJ. The role of the microbiome in cancer development and therapy. CA Cancer J Clin (2017) 67(4):326–44. 10.3322/caac.21398 PMC5530583 28481406

[31] Robsahm TE Aagnes B Hjartåker A Langseth H Bray FI Larsen IK. Body mass index, physical activity, and colorectal cancer by anatomical subsites: A systematic review and meta-analysis of cohort studies. Eur J Cancer Prev (2013) 22(6):492–505. 10.1097/CEJ.0b013e328360f434 23591454

[32] Chan DSM Lau R Aune D Vieira R Greenwood DC Kampman E. Red and processed meat and colorectal cancer incidence: Meta-analysis of prospective studies. PloS One (2011) 6(6):e20456. 10.1371/journal.pone.0020456 21674008 PMC3108955

[33] Zmora N Suez J Elinav E. You are what you eat: diet, health and the gut microbiota. Nat Rev Gastroenterol Hepatol (2019) 16(1):35–56. 10.1038/s41575-018-0061-2 30262901

[34] Saus E Iraola-Guzmán S Willis JR Brunet-Vega A Gabaldón T. Microbiome and colorectal cancer: Roles in carcinogenesis and clinical potential. Mol Aspects Med (2019) 69:93–106. 10.1016/j.mam.2019.05.001 31082399 PMC6856719

[35] Zeng H. Mechanisms linking dietary fiber, gut microbiota and colon cancer prevention. World J Gastrointest Oncol (2014) 6(2):41–51. 10.4251/wjgo.v6.i2.41 24567795 PMC3926973

[36] Alhinai EA Walton GE Commane DM. The role of the gut microbiota in colorectal cancer causation. Int J Mol Sci (2019) 20(21):5295. 10.3390/ijms20215295 PMC6862640 31653078

[37] Knudsen KEB Lærke HN Hedemann MS Nielsen TS Ingerslev AK Nielsen DSG. Impact of diet-modulated butyrate production on intestinal barrier function and inflammation. Nutrients (2018) 10(10):1499. 10.3390/nu10101499 PMC6213552 30322146

[38] Bäckhed F Fraser CM Ringel Y Sanders ME Sartor RB Sherman PM. Defining a healthy human gut microbiome: Current concepts, future directions, and clinical applications. Cell Host Microbe (2012) 12(5):611–22. 10.1016/j.chom.2012.10.012 23159051

[39] Kuzma J Chmelař D Hájek M Lochmanová A Čižnár I Rozložník M. The role of intestinal microbiota in the pathogenesis of colorectal carcinoma. Folia Microbiol (2020) 34(3):1285–300. 10.1007/s12223-019-00706-2 31001762

[40] Sekirov I Russell SL Caetano M Antunes L Finlay BB. Gut microbiota in health and disease. Physiol Rev (2010) 90(3):859–904. 10.1152/physrev.00045.2009 20664075

[41] O’Hara AM Shanahan F. The gut flora as a forgotten organ. EMBO Rep (2006) 7(7):688–93. 10.1038/sj.embor.7400731 PMC1500832 16819463

[42] Sanyaolu LN Oakley NJ Nurmatov U Dolwani S Ahmed H. Antibiotic exposure and the risk of colorectal adenoma and carcinoma: a systematic review and meta-analysis of observational studies. Colorectal Dis. (2020) 22(8):858–70. 10.1111/codi.14921 31802593

[43] Ferrer-Mayorga G Larriba MJ Crespo P Muñoz A. Mechanisms of action of vitamin D in colon cancer. J Steroid Biochem Mol Biol (2019) 185:1–6. 10.1016/j.jsbmb.2018.07.002 29981368

[44] Al Nabhani Z Dietrich G Hugot JP Barreau F. Nod2: The intestinal gate keeper. PloS Pathogens (2017) 13(3):e1006177. 10.1371/journal.ppat.1006177 28253332 PMC5333895

[45] Kato K Ishida S Tanaka M Mitsuyama E Xiao JZ Odamaki T. Association between functional lactase variants and a high abundance of Bifidobacterium in the gut of healthy Japanese people. PloS One (2018) 13(10):e0206189. 10.1371/journal.pone.0206189 30339693 PMC6195297

[46] Allen A Hutton DA Pearson JP. The MUC2 gene product: A human intestinal mucin. Int J Biochem Cell Biol (1998) 30(7):797–801. 10.1016/S1357-2725(98)00028-4 9722984

[47] Wacklin P Tuimala J Nikkilä J Tims S Mäkivuokko H Alakulppi N. Faecal microbiota composition in adults is associated with the FUT2 gene determining the secretor status. PloS One (2014) 9(4):e94863. 10.1371/journal.pone.0094863 24733310 PMC3986271

[48] Zheng D Liwinski T Elinav E. Interaction between microbiota and immunity in health and disease. Cell Res (2020) 30(6):492–506. 10.1038/s41422-020-0332-7 32433595 PMC7264227

[49] Flanagan L Schmid J Ebert M Soucek P Kunicka T Liska V. Fusobacterium nucleatum associates with stages of colorectal neoplasia development, colorectal cancer and disease outcome. Eur J Clin Microbiol Infect Dis (2014) 33(8):1381–90. 10.1007/s10096-014-2081-3 24599709

[50] Gao R Kong C Ebert L Li H Qu X Liu Z. Mucosa-associated microbiota signature in colorectal cancer. Eur J Clin Microbiol Infect Dis (2017) 36(11):2073–83. 10.1007/s10096-017-3026-4 28600626

[51] Rezasoltani S Sharafkhah M Asadzadeh Aghdaei H Nazemalhosseini Mojarad E Dabiri H Akhavan Sepahi A. Applying simple linear combination, multiple logistic and factor analysis methods for candidate fecal bacteria as novel biomarkers for early detection of adenomatous polyps and colon cancer. J Microbiol Methods (2018) 155:82–8. 10.1016/j.mimet.2018.11.007 30439465

[52] Kumar R Herold JL Schady D Davis J Kopetz S Martinez-Moczygemba M. Streptococcus gallolyticus subsp. gallolyticus promotes colorectal tumor development. PloS Pathog (2017) 13(7):e1006440. 10.1371/journal.ppat.1006440 28704539 PMC5509344

[53] Rezasoltani S Asadzadeh Aghdaei H Dabiri H Akhavan Sepahi A Modarressi MH Nazemalhosseini Mojarad E. The association between fecal microbiota and different types of colorectal polyp as precursors of colorectal cancer. Microb Pathog (2018) 124:244–9. 10.1016/j.micpath.2018.08.035 30142468

[54] Yu J Feng Q Wong SH Zhang D Yi Liang Q Qin Y. Metagenomic analysis of faecal microbiome as a tool towards targeted non-invasive biomarkers for colorectal cancer. Gut (2017) 66(1):70–78. 10.1136/gutjnl-2015-309800 26408641

[55] Gagnière J Raisch J Veziant J Barnich N Bonnet R Buc E. Gut microbiota imbalance and colorectal cancer. World J Gastroenterol (2016) 22(2):501–18. 10.3748/wjg.v22.i2.501 PMC4716055 26811603

[56] Feng Q Liang S Jia H Stadlmayr A Tang L Lan Z. Gut microbiome development along the colorectal adenoma-carcinoma sequence. Nat Commun (2015) 6:6528. 10.1038/ncomms7528 25758642

[57] Ternes D Karta J Tsenkova M Wilmes P Haan S Letellier E. Microbiome in Colorectal Cancer: How to Get from Meta-omics to Mechanism?. Trends Microbiol (2020) 28(5):401–23. 10.1016/j.tim.2020.01.001 32298617

[58] Schwabe RF Jobin C. The microbiome and cancer. Nat Rev Cancer (2013) 13(11):800–12. 10.1038/nrc3610 PMC3986062 24132111

[59] Kostic AD Chun E Robertson L Glickman JN Gallini CA Michaud M. Fusobacterium nucleatum Potentiates Intestinal Tumorigenesis and Modulates the Tumor-Immune Microenvironment. Cell Host Microbe (2013) 14(2):207–15. 10.1016/j.chom.2013.07.007 PMC3772512 23954159

[60] Jans C Boleij A. The road to infection: Host-microbe interactions defining the pathogenicity of Streptococcus bovis/Streptococcus equinus complex members. Front Microbiol (2018) 9:603. 10.3389/fmicb.2018.00603 29692760 PMC5902542

[61] Weitzman MD Weitzman JB. What’s the damage? The impact of pathogens on pathways that maintain host genome integrity. Cell Host Microbe (2014) 15(3):283–94. 10.1016/j.chom.2014.02.010 PMC4501477 24629335

[62] Kosumi K Mima K Baba H Ogino S. Dysbiosis of the gut microbiota and colorectal cancer: the key target of molecular pathological epidemiology. J Lab Precis Med (2018 3:76. 10.21037/jlpm.2018.09.05 30345420 PMC6195365

[63] Hughes DA Bacigalupe R Wang J Rühlemann MC Tito RY Falony G. Genome-wide associations of human gut microbiome variation and implications for causal inference analyses. Nat Microbiol (2020) 5(9):1079–87. 10.1038/s41564-020-0743-8 PMC7610462 32572223

[64] Ma C Chen K Wang Y Cen C Zhai Q Zhang J. Establishing a novel colorectal cancer predictive model based on unique gut microbial single nucleotide variant markers. Gut Microbes (2021) 13(1):1–6. 10.1080/19490976.2020.1869505 PMC7808391 33430705

[65] Hamada T Nowak JA Milner DA Song M Ogino S. Integration of microbiology, molecular pathology, and epidemiology: a new paradigm to explore the pathogenesis of microbiome-driven neoplasms. J Pathol (2019) 247(5):615–28. 10.1002/path.5236 PMC6509405 30632609

[66] Mima K Kosumi K Baba Y Hamada T Baba H Ogino S. The microbiome, genetics, and gastrointestinal neoplasms: the evolving field of molecular pathological epidemiology to analyze the tumor–immune–microbiome interaction. Hum Genet (2020). 10.1007/s00439-020-02235-2 PMC8052267 33180176

[67] Yoon MY Lee K Yoon SS. Protective role of gut commensal microbes against intestinal infections. J Microbiol (2014) 52(12):983–9. 10.1007/s12275-014-4655-2 25467115

[68] Desai MS Seekatz AM Koropatkin NM Kamada N Hickey CA Wolter M. A Dietary Fiber-Deprived Gut Microbiota Degrades the Colonic Mucus Barrier and Enhances Pathogen Susceptibility. Cell (2016) 167(5):1339–53.e21. 10.1016/j.cell.2016.10.043 27863247 PMC5131798

[69] Van der Sluis M De Koning BAE De Bruijn ACJM Velcich A Meijerink JPP Van Goudoever JB. Muc2-Deficient Mice Spontaneously Develop Colitis, Indicating That MUC2 Is Critical for Colonic Protection. Gastroenterology (2006) 131(1):117–29. 10.1053/j.gastro.2006.04.020 16831596

[70] Ocvirk S Sava IG Lengfelder I Lagkouvardos I Steck N Roh JH. Surface-Associated Lipoproteins Link Enterococcus faecalis Virulence to Colitogenic Activity in IL-10-Deficient Mice Independent of Their Expression Levels. PloS Pathog (2015) 11(6):e1004911. 10.1371/journal.ppat.1004911 26067254 PMC4466351

[71] Sobieszczańska B Duda-Madej A Turniak M Franiczek R Kasprzykowska U Duda AK. Invasive properties, adhesion patterns and phylogroup profiles among Escherichia Coli strains isolated from children with inflammatory bowel disease. Adv Clin Exp Med (2012) 21(5):591–9. 23356195

[72] Han YW Shi W Huang GTJ Kinder Haake S Park NH Kuramitsu H. Interactions between periodontal bacteria and human oral epithelial cells: Fusobacterium nucleatum adheres to and invades epithelial cells. Infect Immun (2000) 68(6):3140–6. 10.1128/IAI.68.6.3140-3146.2000 PMC97547 10816455

[73] Rubinstein MR Wang X Liu W Hao Y Cai G Han YW. Fusobacterium nucleatum Promotes Colorectal Carcinogenesis by Modulating E-Cadherin/β-Catenin Signaling via its FadA Adhesin. Cell Host Microbe (2013) 14(2):195–206. 10.1016/j.chom.2013.07.012 23954158 PMC3770529

[74] Wu S Lim KC Huang J Saidi RF Sears CL. Bacteroides fragilis enterotoxin cleaves the zonula adherens protein, E-cadherin. Proc Natl Acad Sci USA (1998) 95(25):14979–84. 10.1073/pnas.95.25.14979 PMC24561 9844001

[75] Hwang S Gwon S-Y Kim MS Lee S Rhee K-J. Bacteroides fragilis Toxin Induces IL-8 Secretion in HT29/C1 Cells through Disruption of E-cadherin Junctions. Immune Netw (2013) 13(5):213–7. 10.4110/in.2013.13.5.213 PMC3817303 24198747

[76] Arthur JC Perez-Chanona E Mühlbauer M Tomkovich S Uronis JM Fan TJ. Intestinal inflammation targets cancer-inducing activity of the microbiota. Science (2012) 338(6103):120–3. 10.1126/science.1224820 PMC3645302 22903521

[77] Derrien M Collado MC Ben-Amor K Salminen S De Vos WM. The mucin degrader Akkermansia muciniphila is an abundant resident of the human intestinal tract. Appl Environ Microbiol (2008) 106:171–81. 10.1128/AEM.01226-07 PMC2258631 18083887

[78] Derrien M Belzer C de Vos WM. Akkermansia muciniphila and its role in regulating host functions. Microbial Pathogenesis (2017) 106:171–81. 10.1016/j.micpath.2016.02.005 26875998

[79] Meng X Zhang J Wu H Yu D Fang X. Akkermansia muciniphila aspartic protease amuc_1434* Inhibits human colorectal cancer LS174T cell viability via TRAIL-mediated apoptosis pathway. Int J Mol Sci (2020) 21(9):3385. 10.3390/ijms21093385 PMC7246985 32403433

[80] Lungulescu CV Răileanu S Afrem G Ungureanu BS Florescu DV Gheonea IA. Histochemical and immunohistochemical study of mucinous rectal carcinoma. J Med Life (2017) 10(2):139–43. PMC5467255 28616090

[81] Ottman N Reunanen J Meijerink M Pietila TE Kainulainen V Klievink J. Pili-like proteins of Akkermansia muciniphila modulate host immune responses and gut barrier function. PloS One (2017) 12(3):e0173004. 10.1371/journal.pone.0173004 28249045 PMC5332112

[82] Plovier H Everard A Druart C Depommier C Van Hul M Geurts L. A purified membrane protein from Akkermansia muciniphila or the pasteurized bacterium improves metabolism in obese and diabetic mice. Nat Med (2017) 23(1):107–13. 10.1038/nm.4236 27892954

[83] VigsnÆs LK Brynskov J Steenholdt C Wilcks A Licht TR. Gram-negative bacteria account for main differences between faecal microbiota from patients with ulcerative colitis and healthy controls. Benef Microbes (2012) 3(4):287–97. 10.3920/BM2012.0018 22968374

[84] James SL Christophersen CT Bird AR Conlon MA Rosella O Gibson PR. Abnormal fibre usage in UC in remission. Gut (2015) 64(4):562–70. 10.1136/gutjnl-2014-307198 25037189

[85] Png CW Lindén SK Gilshenan KS Zoetendal EG McSweeney CS Sly LI. Mucolytic bacteria with increased prevalence in IBD mucosa augment in vitro utilization of mucin by other bacteria. Am J Gastroenterol (2010) 105(11):2420–8. 10.1038/ajg.2010.281 20648002

[86] Ding C Tang W Fan X Wu G. Intestinal microbiota: A novel perspective in colorectal cancer biotherapeutics. OncoTargets Ther (2018) 11:4797–810. 10.2147/OTT.S170626 PMC6097518 30147331

[87] Wu CC Lu YZ Wu LL Yu LC. Role of myosin light chain kinase in intestinal epithelial barrier defects in a rat model of bowel obstruction. BMC Gastroenterol (2010) 10:39. 10.1186/1471-230X-10-39 20403206 PMC2868795

[88] Buchheister S Buettner M Basic M Noack A Breves G Buchen B. CD14 Plays a Protective Role in Experimental Inflammatory Bowel Disease by Enhancing Intestinal Barrier Function. Am J Pathol (2017) 187(5):1106–20. 10.1016/j.ajpath.2017.01.012 28411859

[89] Liu X Cheng Y Shao L Ling Z Huang Y. Alterations of the Predominant Fecal Microbiota and Disruption of the Gut Mucosal Barrier in Patients with Early-Stage Colorectal Cancer. BioMed Res Int (2020) 2020:2948282. 10.1155/2020/2948282 32280686 PMC7114766

[90] Wiercinska-Drapalo A Jaroszewicz J Siwak E Pogorzelska J Prokopowicz D. Intestinal fatty acid binding protein (I-FABP) as a possible biomarker of ileitis in patients with ulcerative colitis. Regul Pept (2008) 147(1-3):25–8. 10.1016/j.regpep.2007.12.002 18201778

[91] Malíčková K Francová I Lukáš M Kolář M Králíková E Bortlík M. Fecal zonulin is elevated in Crohn’s disease and in cigarette smokers. Pract Lab Med (2017) 9:39–44. 10.1016/j.plabm.2017.09.001 PMC5633835 29034305

[92] Tripathi A Lammers KM Goldblum S Shea-Donohue T Netzel-Arnett S Buzza MS. Identification of human zonulin, a physiological modulator of tight junctions, as prehaptoglobin-2. Proc Natl Acad Sci USA (2009) 106(39):16799–804. 10.1073/pnas.0906773106 PMC2744629 19805376

[93] Bertiaux-Vandaële N Youmba SB Belmonte L Lecleire S Antonietti M Gourcerol G. The expression and the cellular distribution of the tight junction proteins are altered in irritable bowel syndrome patients with differences according to the disease subtype. Am J Gastroenterol (2011) 106(12):2165–73. 10.1038/ajg.2011.257 22008894

[94] Umeda K Ikenouchi J Katahira-Tayama S Furuse K Sasaki H Nakayama M. ZO-1 and ZO-2 Independently Determine Where Claudins Are Polymerized in Tight-Junction Strand Formation. Cell (2006) 126(4):741–54. 10.1016/j.cell.2006.06.043 16923393

[95] Kinugasa T Akagi Y Yoshida T Ryu Y Shiratuchi I Ishibashi N. Increased claudin-1 protein expression contributes to tumorigenesis in ulcerative colitis-associated colorectal cancer. Anticancer Res (2010) 30(8):3181–6. 10.1016/S0016-5085(11)61434-0 20871038

[96] Pope JL Bhat AA Sharma A Ahmad R Krishnan M Washington MK. Claudin-1 regulates intestinal epithelial homeostasis through the modulation of Notch-signalling. Gut (2014) 63(4):622–34. 10.1136/gutjnl-2012-304241 PMC4083824 23766441

[97] Zhao H Yu H Martin TA Zhang Y Chen G Jiang WG. Effect of junctional adhesion molecule-2 expression on cell growth, invasion and migration in human colorectal cancer. Int J Oncol (2016) 48(3):929–36. 10.3892/ijo.2016.3340 PMC4750534 26782073

[98] Blair SA Kane SV Clayburgh DR Turner JR. Epithelial myosin light chain kinase expression and activity are upregulated in inflammatory bowel disease. Lab Investig (2006) 86(2):191–201. 10.1038/labinvest.3700373 16402035

[99] Kong SY Tran HQ Gewirtz AT McKeown-Eyssen G Fedirko V Romieu I. Serum endotoxins and flagellin and risk of colorectal cancer in the European prospective investigation into cancer and nutrition (EPIC) cohort. Cancer Epidemiol Biomarkers Prev (2016) 25(2):291–301. 10.1158/1055-9965.EPI-15-0798 26823475 PMC5576525

[100] Guo S Al-Sadi R Said HM Ma TY. Lipopolysaccharide causes an increase in intestinal tight junction permeability in vitro and in vivo by inducing enterocyte membrane expression and localization of TLR-4 and CD14. Am J Pathol (2013) 182(2):375–87. 10.1016/j.ajpath.2012.10.014 PMC3562736 23201091

[101] Tabung FK Birmann BM Epstein MM Martínez-Maza O Breen EC Wu K. Influence of Dietary Patterns on Plasma Soluble CD14, a Surrogate Marker of Gut Barrier Dysfunction. Curr Dev Nutr (2017) 1(11):e001396. 10.3945/cdn.117.001396 29595830 PMC5867900

[102] Bindels LB Neyrinck AM Loumaye A Catry E Walgrave H Cherbuy C. Increased gut permeability in cancer cachexia: Mechanisms and clinical relevance. Oncotarget (2018) 9(26):18224–38. 10.18632/oncotarget.24804 PMC5915068 29719601

[103] Grivennikov SI Wang K Mucida D Stewart CA Schnabl B Jauch D. Adenoma-linked barrier defects and microbial products drive IL-23/IL-17-mediated tumour growth. Nature (2012) 491(7423):254–8. 10.1038/nature11465 PMC3601659 23034650

[104] Bruewer M Luegering A Kucharzik T Parkos CA Madara JL Hopkins AM. Proinflammatory Cytokines Disrupt Epithelial Barrier Functionby Apoptosis-Independent Mechanisms. J Immunol (2003) 171(11):6164–72. 10.4049/jimmunol.171.11.6164 14634132

[105] Niccolai E Baldi S Ricci F Russo E Nannini G Menicatti M. Evaluation and comparison of short chain fatty acidscomposition in gut diseases. World J Gastroenterol(2019) 25(36):5543–58. 10.3748/wjg.v25.i36.5543 PMC6767983 31576099

[106] Weir TL Manter DK Sheflin AM Barnett BA Heuberger AL Ryan EP. Stool Microbiome and Metabolome Differences betweenColorectal Cancer Patients and Healthy Adults. PloS One (2013) 8(8):e70803. 10.1371/journal.pone.0070803 23940645 PMC3735522

[107] Wang S Dong W Liu L Xu M Wang Y Liu T. Interplay between bile acids and the gut microbiota promotesintestinal carcinogenesis. Mol Carcinog (2019)58(7):1155–67. 10.1002/mc.22999 PMC6593857 30828892

[108] Assa A Vong L Pinnell LJ Avitzur N Johnson-Henry KC Sherman PM. Vitamin D deficiency promotes epithelial barrier dysfunctionand intestinal inflammation. J Infect Dis(2014) 210(8):1296–305. 10.1093/infdis/jiu235 24755435

[109] Bjarnason I. The use of fecal calprotectin in inflammatory boweldisease. Gastroenterol Hepatol (2017)13(1):53–56. PMC5390326 28420947

[110] Lau E Marques C Pestana D Santoalha M Carvalho D Freitas D. The role of I-FABP as a biomarker of intestinal barrierdysfunction driven by gut microbiota changes in obesity. Nutr Metab (2016) 13:31. 10.1186/s12986-016-0089-7 PMC4851788 27134637

[111] Bottasso Arias NM García M Bondar C Guzman L Redondo A Chopita N. Expression pattern of fatty acid binding proteins in celiac disease enteropathy. Mediators Inflamm (2015) 2015:738563. 10.1155/2015/738563 26346822 PMC4540995

[112] Linsalata M Riezzo G D’Attoma B Clemente C Orlando A Russo F. Noninvasive biomarkers of gut barrier function identify two subtypes of patients suffering from diarrhoea predominant-IBS: A case-control study. BMC Gastroenterol (2018) 18(1):167. 10.1186/s12876-018-0888-6 30400824 PMC6219148

[113] Pelsers MMAL Namiot Z Kisielewski W Namiot A Januszkiewicz M Hermens WT. Intestinal-type and liver-type fatty acid-binding protein in the intestine. Tissue distribution and clinical utility. Clin Biochem (2003) 36(7):529–35. 10.1016/S0009-9120(03)00096-1 14563446

[114] Sturgeon C Fasano A. Zonulin, a regulator of epithelial and endothelial barrier functions, and its involvement in chronic inflammatory diseases. Tissue Barriers (2016) 4(4):e1251384. 10.1080/21688370.2016.1251384 28123927 PMC5214347

[115] Fasano A Not T Wang W Uzzau S Berti I Tommasini A. Zonulin, a newly discovered modulator of intestinal permeability, and its expression in coeliac disease. Lancet (2000) 355(9214):1518–9. 10.1016/S0140-6736(00)02169-3 10801176

[116] El Asmar R Paningrahi P Bamford P Berti I Not T Catassi C. Zonulin is involved in the impairment of the gut barrier function following small intestinal bacterial colonization. Gastroenterology (2000) 118(4):A815. 10.1016/s0016-5085(00)85402-5 12404235

[117] El Asmar R Panigrahi P Bamford P Berti I Not T Coppa GV. Host-dependent zonulin secretion causes the impairment of thesmall intestine barrier function after bacterial exposure.Gastroenterology (2002) 123(5):1607–15. 10.1053/gast.2002.36578 12404235

[118] Zeisel MB Dhawan P Baumert TF. Tight junction proteins in gastrointestinal and liverdisease. Gut (2019) 68(3):547–61. 10.1136/gutjnl-2018-316906 PMC6453741 30297438

[119] Pope JL Ahmad R Bhat AA Washington MK Singh AB Dhawan P. Claudin-1 overexpression in intestinal epithelial cellsenhances susceptibility to adenamatous polyposis coli-mediated colon tumorigenesis. Mol Cancer (2014) 13:167. 10.1186/1476-4598-13-167 24997475 PMC4105545

[120] Su L Nalle SC Shen L Turner ES Singh G Breskin LA. TNFR2 activates mlck-dependent tight junction dysregulation to cause apoptosis-mediated barrier loss and experimental colitis. Gastroenterology (2013) 145(2):407–15. 10.1053/j.gastro.2013.04.011 PMC3722284 23619146

[121] Zhao L Luo L Jia W Xiao J Huang G Tian G. Serum diamine oxidase as a hemorrhagic shock biomarker in a rabbit model. PloS One (2014) 9(8):e102285. 10.1371/journal.pone.0102285 25144315 PMC4140717

[122] O’Keefe SJD Ou J Aufreiter S O’Connor D Sharma S Sepulveda J. Products of the Colonic Microbiota Mediate the Effects of Diet on Colon Cancer Risk. J Nutr (2009) 139(11):2044–8. 10.3945/jn.109.104380 PMC6459055 19741203

[123] Vipperla K O’Keefe SJ. The microbiota and its metabolites in colonic mucosal health and cancer risk. Nutr Clin Practice (2012) 27(5):624–35. 10.1177/0884533612452012 22868282

[124] Ridlon JM Kang DJ Hylemon PB. Bile salt biotransformations by human intestinal bacteria. J Lipid Res (2006) 47(2):241–59. 10.1194/jlr.R500013-JLR200 16299351

[125] Ajouz H Mukherji D Shamseddine A. Secondary bile acids: An underrecognized cause of colon cancer. World J Surg Oncol (2014) 12:164. 10.1186/1477-7819-12-164 24884764 PMC4041630

[126] Nguyen TT Lian S Ung TT Xia Y Han JY Do Jung Y. Lithocholic Acid Stimulates IL-8 Expression in Human Colorectal Cancer Cells Via Activation of Erk1/2 MAPK and Suppression of STAT3 Activity. J Cell Biochem (2017) 118(9):2958–67. 10.1002/jcb.25955 28247965

[127] Cao H Luo S Xu M Zhang Y Song S Wang S. The secondary bile acid, deoxycholate accelerates intestinal adenoma–adenocarcinoma sequence in Apc min/+ mice through enhancing Wnt signaling. Fam Cancer (2014) 13(4):563–71. 10.1007/s10689-014-9742-3 25106466

[128] Cao H Xu M Dong W Deng B Wang S Zhang Y. Secondary bile acid-induced dysbiosis promotes intestinal carcinogenesis. Int J Cancer (2017) 140(11):2545–56. 10.1002/ijc.30643 28187526

[129] Kim J Artinyan A Mailey B Mailey S Lee W McKenzie S. An interaction of race and ethnicity with socioeconomic status in rectal cancer outcomes. Ann Surg (2011) 253(4)647–54. 10.1097/SLA.0b013e3182111102 21475002

[130] Yothers G Sargent DJ Wolmark N Goldberg RM O’Connell MJ Benedetti JK. Outcomes among black patients with stage II and III colon cancer receiving chemotherapy: An analysis of ACCENT adjuvant trials. J Natl Cancer Inst (2011) 103(20):1498–506. 10.1093/jnci/djr310 PMC3196480 21997132

[131] Lanier AP Day GE Kelly JJ Provost E. Disparities in cancer mortality among Alaska Native people, 1994-2003. Alaska Med (2008) 49(4):120–5. 18491804

[132] Hester CM Jala VR Langille MGI Umar S Greiner KA Haribabu B. Fecal microbes, short chain fatty acids, and colorectal cancer across racial/ethnic groups. World J Gastroenterol (2015) 21(9):2759–69. 10.3748/wjg.v21.i9.2759 PMC4351229 25759547

[133] Hughes DJ Fedirko V Jenab M Schomburg L Méplan C Freisling H. Selenium status is associated with colorectal cancer risk in the European prospective investigation of cancer and nutrition cohort. Int J Cancer (2015) 136(5):1149–61. 10.1002/ijc.29071 25042282

[134] McCullough ML Zoltick ES Weinstein SJ Fedirko V Wang M Cook NR. Circulating Vitamin D and colorectal cancer risk: An international pooling project of 17 cohorts. J Natl Cancer Inst (2019) 111(2):158–69. 10.1093/jnci/djy087 PMC6376911 29912394

[135] Stepien M Jenab M Freisling H Becker NP Czuban M Tjø;nneland. Pre-diagnostic copper and zinc biomarkers and colorectal cancer risk in the European Prospective Investigation into Cancer and Nutrition cohort. Carcinogenesis (2017) 38(7):699–707. 10.1093/carcin/bgx051 28575311

[136] Yamamoto EA Jørgensen TN. Relationships Between Vitamin D, Gut Microbiome, and Systemic Autoimmunity. Front Immunol (2020) 10:3141. 10.3389/fimmu.2019.03141 32038645 PMC6985452

[137] Zhai Q Cen S Li P Tian F Zhao J Zhang H. Effects of Dietary Selenium Supplementation on Intestinal Barrier and Immune Responses Associated with Its Modulation of Gut Microbiota. Environ Sci Technol Lett (2018) 5(12):724–30. 10.1021/acs.estlett.8b00563

[138] Zhong W McClain CJ Cave M Kang YJ Zhou Z. The role of zinc deficiency in alcohol-induced intestinal barrier dysfunction. Am J Physiol - Gastrointest Liver Physiol (2010) 298(5):G625–33. 10.1152/ajpgi.00350.2009 PMC2867425 20167873

[139] Zhang Y Wu S Sun J. Vitamin D, vitamin D receptor and tissue barriers. Tissue Barriers (2013) 1(1):e23118. 10.4161/tisb.23118 24358453 PMC3865708

[140] Kong J Zhang Z Musch MW Ning G Sun J Hart J. Novel role of the vitamin D receptor in maintaining the integrity of the intestinal mucosal barrier. Am J Physiol - Gastrointest Liver Physiol (2007) 294(1):G208–16. 10.1152/ajpgi.00398.2007 17962355

[141] Cavaillon JM. Exotoxins and endotoxins: Inducers of inflammatory cytokines. Toxicon (2018) 149:45–53. 10.1016/j.toxicon.2017.10.016 29056305

[142] González-Sarrías A Núñez-Sánchez MA Ávila-Gálvez MW Monedero-Saiz T Rodríguez-Gil FJ Martínez-Díaz F. Consumption of pomegranate decreases plasma lipopolysaccharide-binding protein levels, a marker of metabolic endotoxemia, in patients with newly diagnosed colorectal cancer: A randomized controlled clinical trial. Food Funct (2018) 9(5):2617–22. 10.1039/c8fo00264a 29770393

[143] Sanders CJ Yu Y Moore DA Williams IR Gewirtz AT. Humoral Immune Response to Flagellin Requires T Cells and Activation of Innate Immunity. J Immunol (2006) 177(5):2810–8. 10.4049/jimmunol.177.5.2810 16920916

[144] Girón-González JA Moral FJ Elvira J García-Gil D Guerrero F Gavilán I. Consistent production of a higher T(H)1:T(H)2 cytokine ratio by stimulated T cells in men compared with women. Eur J Endocrinol (2000) 143(1):31–6. 10.1530/eje.0.1430031 10870028

[145] Klein SL. The effects of hormones on sex differences in infection: From genes to behavior. Neurosci Biobehav Rev (2000) 24(6):627–38. 10.1016/S0149-7634(00)00027-0 10940438

[146] Pellegrini P Contasta I Del Beato T Ciccone F Berghella AM. Gender-specific cytokine pathways, targets, and biomarkers for the switch from health to adenoma and colorectal cancer. Clin Dev Immunol (2011) 2011:819724. 10.1155/2011/819724 22235223 PMC3253453

[147] Xiao S Zhao L. Gut microbiota-based translational biomarkers to prevent metabolic syndrome via nutritional modulation. FEMS Microbiol Ecol (2014) 87(2):303–14. 10.1111/1574-6941.12250 PMC4262049 24219358

[148] Schumann RR Leong SR Flaggs GW Gray PW Wright SD Mathison JC. Structure and function of lipopolysaccharide binding protein. Science (1990) 249(4975):1429–31. 10.1126/science.2402637 2402637

[149] Kamada N Hisamatsu T Okamoto S Chinen H Kobayashi T Sato T. Unique CD14+ intestinal macrophages contribute to the pathogenesis of Crohn disease via IL-23/IFN-γ axis. J Clin Invest (2008) 118(6):2269–80. 10.1172/JCI34610 PMC2391067 18497880

[150] Seth A Yan F Polk DB Rao RK. Probiotics ameliorate the hydrogen peroxide-induced epithelial barrier disruption by a PKC- And MAP kinase-dependent mechanism. Am J Physiol - Gastrointest Liver Physiol (2008) 294(4):G1060–9. 10.1152/ajpgi.00202.2007 PMC2653458 18292183

[151] Ewaschuk J Endersby R Thiel D Diaz H Backer J Ma N. Probiotic bacteria prevent hepatic damage and maintain colonic barrier function in a mouse model of sepsis. Hepatology (2007) 46(3):841–50. 10.1002/hep.21750 17659579

[152] Butt J Romero-Hernández B Pérez-Gómez B Willhauck-Fleckenstein M Holzinger D Martin V. Association of Streptococcus gallolyticus subspecies gallolyticus with colorectal cancer: Serological evidence. Int J Cancer (2016) 138(7):1670–9. 10.1002/ijc.29914 26537841

[153] Garza-González E Ríos M Bosques-Padilla FJ Francois F Cho I Gonz´lez GM. Immune response against Streptococcus gallolyticus in patients with adenomatous polyps in colon. Int J Cancer (2012) 131(10):2294–9. 10.1002/ijc.27511 22377818

[154] Butt J Jenab M Willhauck-Fleckenstein M Michel A Pawlita M Kyrø C. Prospective evaluation of antibody response to Streptococcus gallolyticus and risk of colorectal cancer. Int J Cancer (2018) 143(2):245–52. 10.1002/ijc.31283 29377173

[155] Butt J Blot WJ Teras LR Visvanathan K Le Marchand L Haiman CA. Antibody responses to Streptococcus Gallolyticus subspecies Gallolyticus proteins in a large prospective colorectal cancer cohort consortium. Cancer Epidemiol Biomarkers Prev (2018) 27(10):1186–94. 10.1158/1055-9965.EPI-18-0249 PMC6170691 30038049

[156] Butt J Varga MG Blot WG Teras L Visvanathan K Le Marchand L. Serologic Response to Helicobacter pylori Proteins Associated With Risk of Colorectal Cancer Among Diverse Populations in the United States. Gastroenterology (2019) 156(1):175–86.e2. 10.1053/j.gastro.2018.09.054 30296434 PMC6309494

[157] Butt J Jenab M Pawlita M Tjønneland A Kyrø C Boutron-Ruault M-C. Antibody Responses to Helicobacter pylori and Risk of Developing Colorectal Cancer in a European Cohort. Cancer Epidemiol Biomarkers Prev (2020) 29(7):1475–81. 10.1158/1055-9965.epi-19-1545 32332031

